# Synergistic effect of cold atmospheric plasma and methylene blue loaded nano micelles on treating human glioblastoma cells: An in vitro and molecular dynamics study

**DOI:** 10.22038/ijbms.2024.79858.17304

**Published:** 2025

**Authors:** Elahe Ahmadi, Armin Imanparast, Mehdi Hoseini, Shahrokh Naseri, Samaneh Soudmand Salarabadi, Ameneh Sazgarnia

**Affiliations:** 1 Medical Physics Research Center, Basic Sciences Research Institute, Mashhad University of Medical Sciences, Mashhad, Iran; 2 Department of Medical Physics, Faculty of Medicine, Mashhad University of Medical Sciences, Mashhad, Iran; 3 Department of Radiology Technology, School of Paramedical Sciences, Torbat Heydarieh University of Medical Sciences, Torbat Heydarieh, Iran

**Keywords:** Cold temperature, Glioblastoma, Methylene blue, Molecular dynamics – simulation, Photochemotherapy, Reactive oxygen species

## Abstract

**Objective(s):**

One of the most recent cancer treatment methods is cold atmospheric plasma (CAP), which destroys cancer cells without affecting healthy cells. Also, the created photons in the CAP flame can be used to excite a proper photosensitizing agent (PS). Therefore, using nano micelle systems containing a proper photosensitizer may be beneficial in raising the treatment efficacy of CAP. In this study, we utilized molecular dynamics (MD) simulation to optimize a nano micellar system containing methylene blue to take advantage of the induced photodynamic effect of a CAP generator with helium gas on a glioblastoma cell line.

**Materials and Methods:**

Some micelle properties were first determined and optimized by MD with GROMACS software. Then, micelles containing methylene blue (Micelle-MB) and free methylene blue (MB) at various concentrations were prepared. Singlet oxygen dosimetry using 1,3-diphenylisobenzofuran (DPBF)was performed in the presence and absence of Micelle-MB and MB. Subsequently, the cytotoxicity of MB and Micelle-MB was evaluated on U87-MG cancer cells, and their half-maximal inhibitory concentrations (IC_50_) were determined. After 48 hr of treatment, the percentage of cell survival was determined using the MTT test. The experiments were repeated at least three times. The synergy index was selected to compare the results.

**Results:**

Treatment with CAP and MB reduced the survival rate compared to the PS-free group with CAP. Results of singlet oxygen dosimetry showed that Micelle-MB might be more efficient in producing ROS. CAP treatment with Micelle-MB resulted in more cell death than free MB. In addition, cell viability decreased in Micelle-MB groups with increasing irradiation time in the three investigated irradiation times.

**Conclusion:**

Using Micelle-MB in the CAP treatment improves treatment efficiency in the U87-MG cell line.

## Introduction

Cancer development can arise from abnormal cell proliferation across various cell types in the body, and there are significant variations in tumor types and treatment responses (1). Among them, Glioblastoma multiforme (GBM) is a high-grade tumor and the most prevalent malignant primary tumor of the brain (2) that is resistant to conventional therapy (Surgery, chemotherapy, and radiotherapy) due to the presence of glioma stem cells (GSCs) that can survive in the microenvironment (3, 4). 

Photodynamic therapy (PDT) is a newly developed treatment option in cancer therapy that uses light, PSs, and oxygen to produce reactive oxygen species (ROS) to eliminate tumor regions specifically. Despite current cancer treatments, PDT can cure microinvasions and protect sensitive brain areas. This advantage over Common cancer treatments can improve patient outcomes (5). If, before performing PDT, it is possible to increase the amount of photosensitizer accumulation in the tumor cell, high treatment efficiency can be expected. The tumor affinity PS undergoes a two-state transition when exposed to a specific light wavelength. The tumor-killing action is caused by the ROS produced due to the energy conversion upon returning to the ground state (3). PDT can cause local hypoxia, which may result in resistance and worse therapeutic outcomes (6). Therefore, providing new treatment strategies that can cover the limitations of PDT is one of the most attractive research topics in preclinical studies.

Among the techniques that can play an effective role in improving the performance of PDT is the induction of photodynamic therapy by cold atmospheric plasma (CAP). Plasma, often considered the fourth state of matter alongside solids, liquids, and gases, is a unique state. It comprises ionized gas, a mix of positive and negative charged particles (ions and electrons), neutral atoms, reactive molecules, and even light particles. In combination treatments in the presence of CAP, in addition to producing ROS and free radicals, emitted light in the CAP flame can induce PDT effects (7). CAP selectively targets and kills cancer cells, making it the focus of attention, which can improve treatment efficacy (8, 9). The selective anticancer mechanism of this modality is due to the abundance of aquaporins in the membrane of cancer cells (10). One theory suggests that aquaporin transporters and reduced cholesterol levels in the membranes of cancer cells, as opposed to non-cancerous cells, enhance the penetration of ROS through the cancer cell membrane, likely through lipid peroxidation. Consequently, more ROS produced by plasma is delivered to cancer cells, leading to increased cell death (11). It has been shown that CAP can cause injuries such as necrosis, cell detachment, apoptosis, and disruption of the S phase in tumor cells via plasma-generated reactive oxide and nitrogen species (RONS). Plus, utilizing CAP, which contains a high concentration of ROS, can overcome the hypoxia challenge in photodynamic therapy (7).

The photodynamic effect induced by CAP (Plasma-induced PDT) is based on the stimulation of photosensitizers using plasma flame photons (7). Many optical sensitizers used in PDAs have weaknesses that reduce their efficiency. One of these photosensitizers is Methylene Blue (MB), which has medicinal properties. MB has low toxicity and no side effects and also has the potential to treat a wide range of malignant and non-malignant disorders (12). MB has an absorption peak at 660 nm, which is used to excite MB in PDT, demonstrating deep tissue penetration properties due to its proximity to the near-infrared window. One of the most important limiting factors of methylene blue in PDT is its inefficient cellular uptake, reinforcing the need to design a suitable nanocarrier (13). 

Micelles are among the most exciting nanocarriers for hydrophobic/hydrophilic drugs in nanomedicine studies. Micelles are obtained from the self-assembly of amphiphilic molecules above the critical micelle concentration (CMC). These carriers can improve the sustained and controlled release of macromolecules, the chemical stability and production of encapsulated molecules, the pharmacokinetics of drugs, and tissue distribution (14). Micelles are usually spherical (15), and the micelle’s dimensions range from 10 to 100 nanometers (16). The concept of HLB number (hydrophilic-lipophilic balance) was introduced as a measure for the polar characteristics of surfactants. HLB is one of the parameters that help to choose suitable surfactants as emulsifying agents. The ratio of the weight percentage of hydrophilic groups to the weight percentage of lipophilic groups in the surfactant molecule is called the HLB value (17, 18).

Considering that the PDT induced by CAP on glioblastoma was proven in the study by Shayesteh Ara *et al.* in 2021 (8), PDT with methylene blue (MB) encapsulated in micelles was also conducted by Bharate *et al.* in 2022 in an *in vitro* study on a human esophageal cancer cell line (14). This study examined CAP’s and MB’s inductive effects on a glioblastoma cell line.

The restricted availability of experimental facilities and the high cost of nanobiological experimental studies encourage researchers to employ simulation methods. Molecular dynamics (MD simulation) facilitates analysis at the molecular and nanoscale. It is a potent tool for gaining a molecular-level understanding of drug interactions, especially aspects not easily accessible through conventional experimental methods. MD simulations provide insights into the spatial disposition and patterns of drug molecules within vehicles, the morphological characteristics of drug-loaded particles, the aggregation tendencies, and the efficacy of drug encapsulation in carriers (18).

This study was a biphasic method. In the first phase, a nanomicelle system containing MB was optimized using molecular dynamic methods with GROMACS software. After that, the PDT induced by CAP was investigated using this optimized nanocomplex as a photosensitizing agent on the glioblastoma cell line.

## Materials and Methods

The execution method was divided into two parts: MD simulation and Experimental Section.

### Molecular dynamics simulation

The Martini force field is a coarse-grained (CG) model designed for conducting MD simulations of biomolecular systems. This force field has been systematically parameterized by accurately reproducing partitioning free energies between polar and apolar phases of numerous chemical compounds. In CG modeling, individual atoms are grouped based on specific criteria to form representative CG interaction sites and MD calculations are then performed between these interaction sites rather than between individual atoms. This technique leads to a significant improvement in computational efficiency. Consequently, monitoring molecular interactions in the time range necessary to understand many phenomena at the time-dependent molecular level is possible (19).

In this research, the Martini Coarse-Grained (CG) Force Field method (20) was used to investigate the MD of the interaction between surfactants. The GROMACS v. 4.6.5 simulation package was used for the MD simulations. VMD (Visual Molecular Dynamics) software was used to visualize the simulation trajectories. For coarse-graining, the Chimera and Gooseview software were utilized. All simulations were performed in a cubic simulation box at a constant temperature of 298 K and a constant pressure of 1 bar. MD simulations of Span80 (SPA) and Tween80 or Polysorbate 80 (P80) surfactant molecules in water at concentrations above the CMC were conducted using a CG force field. In this work, to investigate the self-assembly of surfactants, the aggregation processes of P80 and SPA were initially considered as a function of time for various simulation settings.

*A) Simulation of P80 micelle*

In this study, P80’CG was employed as described in the article by Mobasheri *et al.* (18). P80 was simulated for ten nanoseconds in a cubic box with dimensions of 11.3 and 9611 CG water particles. 

*B) Simulation of SPA micelle*

For this surfactant, several simulations were performed in different boxes with different numbers of CG water particles and SPA molecules due to the lack of a standard CG and aggregation number for simulating a normal micelle. The aggregation number of a micelle refers to the quantity of surfactant molecules that constitute the micelle. The final simulation was performed for 100 nanoseconds, with 3208 CG water particles and 60 SPA.

*C) Simulation of the final structure of micelle*

For this step, several simulations were performed to form a complete micelle. For example, a simulation of 90 SPA molecules, 60 P80 molecules, and 9328 CG water particles was placed in the simulation box.

*D) Simulation of Micelle* *-* *MB*

In this simulation stage, 10 MB molecules and 9219 CG water particles were added to the complete micelle, containing 60 P80s molecules and 90 SPAs molecules.

*Computational analysis of micelles dimensions*

The formula $e=\sqrt[{2}]{1-\frac{c^{2}}{a^{2}}}$ can be used to calculate a micelle’s eccentricity (e), where c and a represent the shortest and longest computed semi-axes, respectively (18). By utilizing the formula, $\mathrm{effective}\mathrm{micelle}\mathrm{radius}=\sqrt[{2}]{\frac{5}{3}}R_{g}$ (18), these numerical measurements provide an accurate representation of the effective micelle radius, incorporating both the micelle core radius (Rc) and the shell thickness (ST). 

### Experimental section

*Chemical*

Fetal bovine serum (FBS) was purchased from Gibco (F7524, USA). Methylene blue, MTT, trypan blue, DMEM, penicillin, trypsin-EDTA, streptomycin, dimethyl sulfoxide (DMSO), DPBF (1,3-Diphenylisobenzofuran), and surfactants (P80, SPA) were obtained from Sigma-Aldrich (St. Louis, MO, USA). All other chemicals were of analytical grade.

*Instruments*

The CAP device employed in this study was purchased from a satellite-based company (Semnan, Iran). It features a copper tube as its central electrode and a copper surface ring as the second electrode. The former is connected to a high-voltage power supply, while the latter is connected to the ground. Helium gas was selected as the carrier gas due to its low breakdown voltage, which facilitates the production of homogeneous and uniform plasma. Specifically, helium gas with a gas flow rate of 4–5 ml/min was utilized to generate a plasma jet. The plasma jet length in the atmospheric ambient was approximately 20 mm. 

*Nano micelles synthesis*

Micelle-MB was prepared using a simple equilibrium method. During stirring, MB and two nonionic surfactants with two completely different hydrophilic–hydrophobic balances (HLBs) were added to the aqueous medium. Surfactants (SPA and P80) and MB were dissolved separately in water to create a uniform solution (a 7.5 mM stock of each surfactant was prepared independently). Then, different percentages of the two surfactants were combined. Following the ratio of two surfactants used in the MD simulation and a series of experimental iterations conducted within the laboratory, it was determined that 2/3 of the ultimate micelle composition consisted of SPA. In this way, a solution of 15 μM MB in micelles was prepared with SPA (HLB of 4.3) with 60% and P80 (HLB of 15) with 40% of the total portion, so an HLB of 8.58 was obtained. The final concentrations of SPA and P80 for the new compounds that were obtained were 2.5 mM and 1.5 mM, respectively. Nonetheless, the concentration for obtaining the polydispersity index (PDI) and the average size of the particles (Z average) was 1.5 mM (SPA) and 1 mM (P80). The final HLB was calculated using the following formula:

Final HLB**= **(SPA_percentage in micelle_ × HLB_SPA_) + (P80_percentage in micelle_ × HLB_P80_) (1)

= (0.6×4.3) + (0.4 ×15) = 8.58

*Nano micelles characterization*

Ultraviolet-visible spectra were obtained using a spectrophotometer (UNICO 2100-UV, China). Also, Dynamic light scattering (DLS) measurements were performed using a nanoparticle analyzer (HORIBA SZ-100, Japan) equipped with a helium-neon (He-Ne) laser (633 nm).

*Assessment of singlet oxygen production*

1,3-diphenylisobenzofuran (DPBF) obtained from Sigma-Aldrich (St. Louis, MO, USA) was used to perform singlet oxygen dosimetry. First, a 0.115mM solution of DPBF in ethanol was prepared. Second, to prepare the dosimeter, different proportions of DPBF and Micelle-MB were made by trial and error, and many aquatic environment tests were conducted since DPBF was destroyed in some high concentrations of micelles. Finally, a ratio of 2/3 DPBF in solution (Water + Micelle-MB + DPBF) was used. Then, the absorption spectrum was recorded at 0.076mM (0.115 mM × 2/3 ).

*Cell culture*

The human glioblastoma U87-MG cell line was obtained from the Pasteur Institute of Iran. In DMEM medium supplemented with 10% FCS and 1% antibiotics, U87-MG cells were grown continuously as monolayers in 75-cm^2^ plastic tissue culture flasks. All cellular cultures were maintained in an incubator with a humidified atmosphere at 37 °C and 5% CO_2_.

*MB and micelle-MB cytotoxicity assay*

The U87-MG cell line was seeded in 96-well plates at 7000 cells/well density and incubated for 48 hr. Then, the cells were incubated with seven different concentrations of MB (0, 2.5, 5, 10, 15, 20, 40 mM) for 4 hr. The cells exposed to MB were subsequently placed in an incubator for 48 hr. Cell viability was assessed using the MTT assay with an ELISA reader (Stat Fax-2100 Awareness, Mountain View, CA, USA). The IC_50_ for MB (concentration of MB which inhibits cell proliferation with 50%) was subsequently determined.

*Experimental treatments*

The U87-MG cell line was cultured in 96-well plates at a density of 7000 cells per well and incubated for 48 hr. Subsequently, the cells were exposed to a 15 µM concentration of MB during incubation. The cellular wells were subjected to the Plasma jet current (Current = 360-380 mA; the plasma jet tip’s distance to the plate’s bottom was 25 mm) for different durations (0, 30, 60, and 90 sec) at ambient temperature. The treated cells were then incubated at 37 °C for 48 hr.

*Cell survival assay*

The viability of U- 87 MG cells was determined using the MTT assay. To perform this assay, following the 48 hr incubation, the culture media was removed, 200 μl of culture media without FBS, and 20 μl of MTT (5 mg/ml in PBS) were added to each well, and the plate was returned to the incubator. After 4 hr, the media was removed from the wells, and the resulting formazan was solubilized with 200 μl of DMSO. Finally, the samples’ optical densities (ODs) were measured at 570 nm against 630 nm using an ELISA reader, and the percentage of cell survival was calculated compared to the control. Subsequently, the exposure time was determined.

*Evaluation indices*

The time of CAP irradiation required a 50% decrease in cell survival (ET_50_) and synergistic ratio (SYN). The synergistic ratio was defined (21) to compare two treatment groups with each other. The synergistic effect was calculated for each agent by quantifying the ratio between observed and anticipated cell death. It is expressed as follows:

The synergistic ratio of MB is the ratio of cell death (CD) caused by radiation in the presence of MB to the total cell death caused by radiation in the presence of each medicinal component individually.

Syn MB+CAP=CD (MB+CAP)CD MB+ CD(CAP) (2)

Syn Micelle+CAP=CD (Micelle+CAP)CD Micelle+ CDCAP (3)

### Statistical analysis

Statistical analysis was performed using SPSS software (version 26). The Kolmogorov-Smirnov goodness-of-fit test was used to check for the normality of data distribution. Due to the non-normal distribution of experimental data, the Mann-Whitney test was used, and *P*<0.05 was considered statistically significant. Excel software was also used to draw survival curves.

## Results

### Molecular dynamics simulation

**Figure 1:**
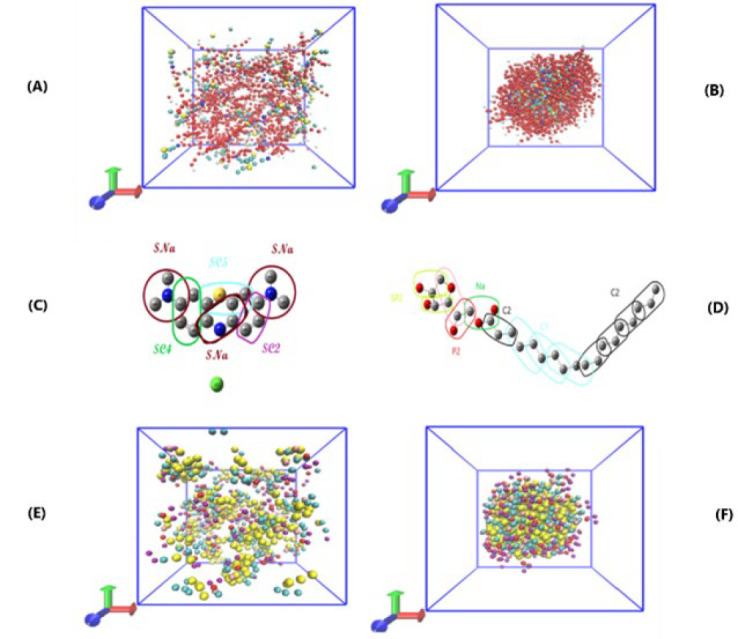
A) Spontaneous formation of micelles containing surfactant P80 at the starting moment, B) Last frame of the micelle, C) Coarse grain of the SPA molecule, D) MB molecule, E) Moment of starting the simulation of micelles containing surfactant SPA, and F) Last frame of the simulation

MB: Methylene blue; SPA: Span80

CG of P80 was done before, so only its simulation in the box was done for ten nanoseconds, and a spherical micelle was created. The formation of micelles in this short period confirms the spontaneous formation of micelles; (Figure 1 (A-B)) illustrates the spontaneous aggregation of P80 molecules.

In this study, several CGs for MB and SPA were designed and displayed in GROMACS software. The best structure was then selected based on some parameters, such as the appropriate distance between grains, and the simulation process progressed with them. (Figure 1C) and (Figure 1D) show the final CG of the MB molecule and SPA molecule, respectively.

Table 1 displays several simulation scenarios involving SPA. As the final simulation of the SPA micelle contained 60 SPA molecules, it was estimated that the aggregation number for SPA is 60. Since the simulation of SPA micelle had not been done before, the duration of 100 nanoseconds was chosen in this study, which is a reasonable time for creating micelles. SPA formed spherical micelles in this short time, indicating its spontaneous formation.)Figure 1 (E-F)( shows the spontaneous aggregation of SPA molecules.

**Figure 2:**
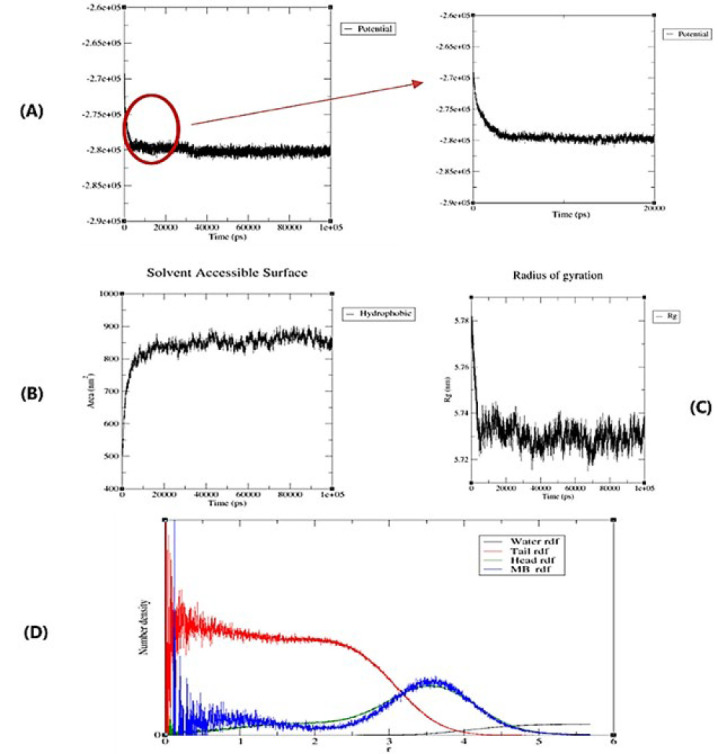
A) Simulation of 60 P80 molecules and 60 SPA molecules, B) Micelles formed with 60 P80 molecules and 90 SPA molecules at zero moments, C) Last frame of the simulation, D to F) Sequence of complete Micelle-MB formation from the start to the last frame of micelle formation, and G) MB was placed in the micelle by removing surfactants from the structure in the final simulation frame (In order to see the position of MB in the micelle, surfactants were not shown in the image)

The SPA aggregation number was estimated to be 60, and the P80 aggregation number was 60. This number of surfactants in both groups formed spherical micelles. Therefore, a complete micelle was expected to be formed without extra surfactants. However, some extra SPA molecules existed in the box (Figure 2A), which demonstrates this, and the aggregation number of SPAs was changed, so the simulation was performed again. The aggregation number of SPA that finally formed complete micelles was 90 molecules. (Figure 2B) shows the micelles formed with 60 P80 molecules (1/3 of all surfactants) and 90 SPA molecules (2/3 of all surfactants) at zero moments. (Figure 2C) shows the last frame of the simulation (100 ns).

 The sequence of Micelle-MB formation from the start to the last frame of micelle formation is shown in (Figure 2 (D-F)). According to (Figure 2G), in the last frame of Micelle-MB, MB is located in the micelle shell, indicating that it has almost no place in the micelle core.

### Analysis of simulation

**Figure 3:**
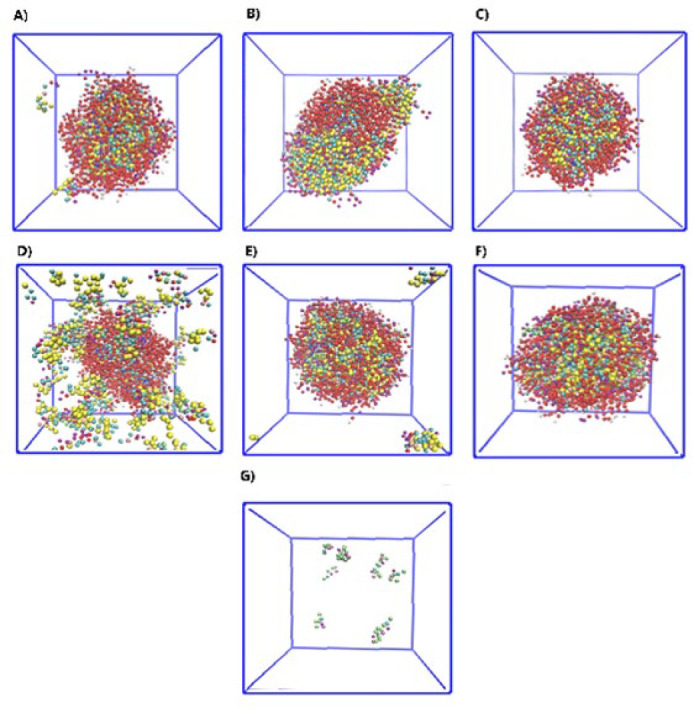
**.** A) Equilibrium energy of the Micelle-MB, B) Accessible Surface Area (SASA), C) Rg diagram of Micelle-MB, and D) Radial distribution function curve of different parts of Micelle-MB

The equilibrium energy of the micelle has been effectively achieved at around 5000 picoseconds, as shown in (Figure 3A). The potential energy declines as the micelle grows, stabilizing when it is in a dynamic equilibrium with the water.

As shown in (Figure 3B), initially, the solvent-accessible surface area (SASA) increases, and then the SASA plot remains constant.

The Micelle-MB form was calculated based on the principal moments of inertia obtained from the simulation results. The average principal axes of inertia ratio are 1.81:1.82:1.83, so the Micelle-MB’s semi-spherical (ellipsoidal) form may be seen. The average Radius of gyration (Rg) analysis of the Micelle-MB is 5.73 nm, and (Figure 3C) shows the Rg diagram. The Rg of the Micelle-MB core and shell is in Table 2. The effective micelle radius is measured to be 7.39 nm.

Figure 3D shows that the micelle core is dry and almost entirely waterless and that the hydrophobic tail has a significant density in the micelle’s center. Based on the radial distribution function of MB, the Micelle-MB shell exhibits the highest MB concentration, a previously validated finding, as depicted in (Figure 2G).

**Figure 4:**
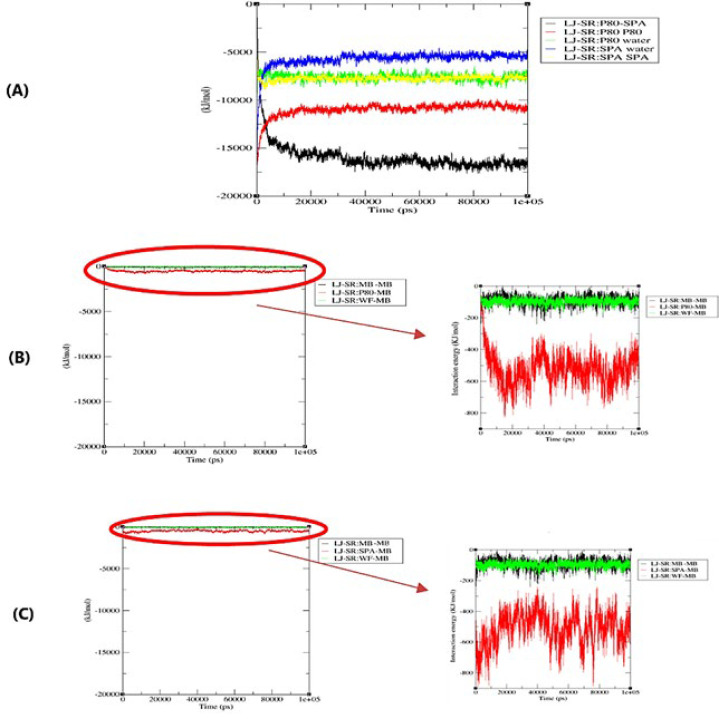
A) Lennard-Jones energy diagram of SPA-SPA, SPA-water, SPA-P80, P80-water, and P80-P80, B) Lennard Jones energy of MB with SPA and water, and C) Lennard Jones Energy MB with P80 and water

MB: Methylene blue; SPA: Span80

Based on (Figure 4(A-C)), the average energy of MB-MB and P80-Water interactions before and after encapsulation remained unchanged. Furthermore, the energy of SPA-Water interactions increased.

### Experimental findings

**Figure 5:**
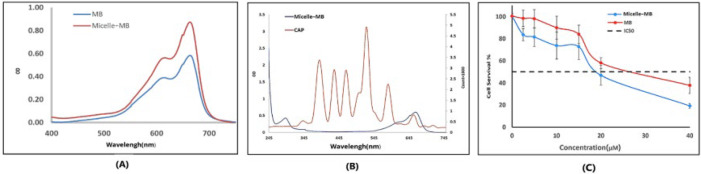
A) Spectra of MB and Micelle-MB at a concentration of 15 μM, B) Overlap of the spectra of plasma and Micelle-MB at a concentration of 15 μM, and C) Cytotoxicity of MB and micelle

MB: Methylene blue; SPA: Span80

*The absorption spectra of MB and Micelle-MB*

)Figure 5A) shows the absorption spectrum of MB and Micelle-MB at 15 μM methylene blue concentration. The absorption peak of MB is lower than that of Micelle-MB.

*The CAP and micelle-MB spectra overlap*

As shown in (Figure 5B), the emission spectrum of the CAP and the absorption spectrum of MB and Micelle-MB overlap at some points. It is expected that the emission peaks of the CAP spectrum in the range of 300-500 nm relatively and in the range of 600–700 nm directly stimulate the absorption peaks of MB.

*Characterization of selected nano micelle system*

The DLS results for micelles with the same surfactant concentrations, but two different PS concentrations are presented in Table 3. It is evident that by raising the concentration of MB from 0.005 to 0.01mM, the nano micelle’s polydispersity index (PDI) and hydrodynamic size decreased.

*Cytotoxicity of the MB and Micelle-MB*

(Figure 5C) displays the outcomes of the dark toxicity test. Micelle-MB has an IC_50_ of 18 μM, while MB has an IC_50_ of 27.5 μM. This issue has increased the uptake, and as a result, the survival percentage has decreased in the Micelle-MB group compared to the MB group.

*Singlet oxygen dosimetry*

**Figure 6:**
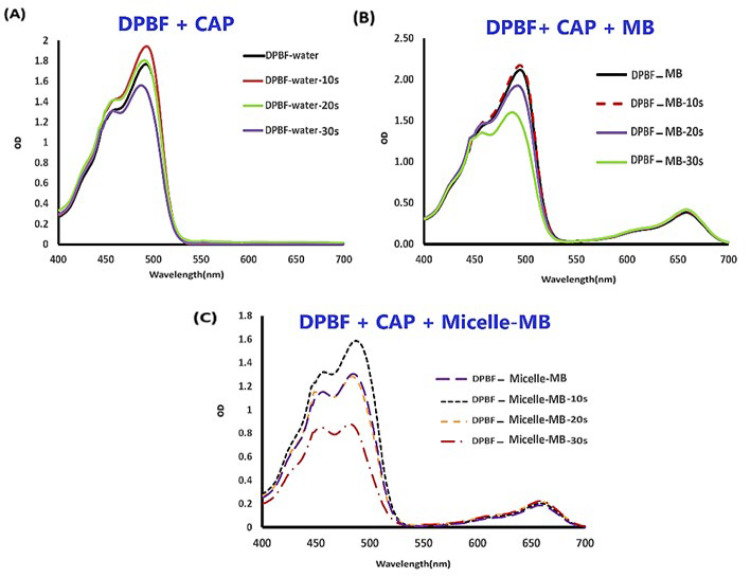
Absorption spectra of MB, Micelles-MB in the presence and absence of DPBF that was exposed to plasma radiation with different time durations

The effect of different CAP irradiation times on the absorption spectrum of DPBF, DPBF+MB, and DPBF+Micelle-MB was measured, which can be seen in (Figure 6). One of the absorption peaks of DPBF is at 490 nm, and that of MB is at 665 nm.

*Cytotoxicity of He-CAP in the presence of MB*

**Figure 7:**
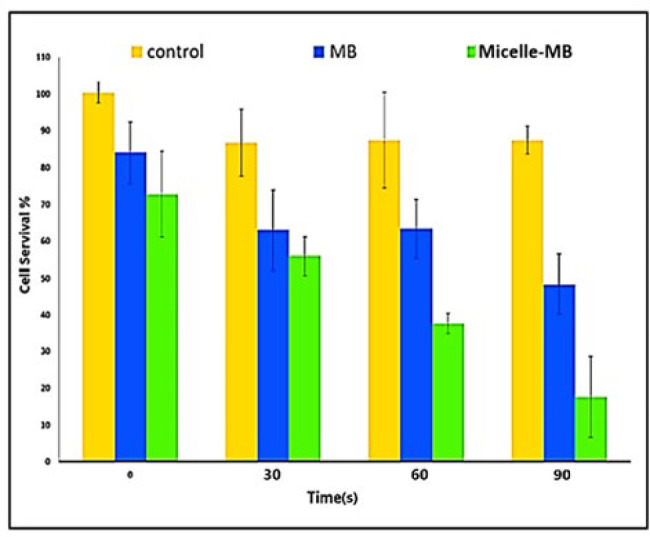
Cytotoxicity of He-CAP in the presence of Micelle-MB at a concentration of 15 μM

Survival percentage chart at irradiation times of 0, 30, 60, and 90 sec in three groups: control, cells incubated with MB, and Micelles-MB can be seen in (Figure 7) at all times, the lowest percentage of survival is related to Micelles-MB, and as the time increases, the survival percentage decreases.

### Synergistic effect

Table 4 displays the SYN of treatment groups exposed to CAP at a concentration of 15 μM MB (IC_30_). Each group experiences an increase in SYN as the radiation time increases. Additionally, in every group, the SYN is higher than one.

## Discussion

In this research, a biphasic study was conducted. In the first phase, we used molecular dynamics to optimize and study the behavior of the Micelle-MB nanomicelle system. In the second phase, we performed photodynamics induced by plasma using the optimized nanomicelle system from the first phase on glioblastoma cancer cells. In the following, we discuss the results of each phase in more detail.

### Molecular dynamic simulation study (Phase #1)

The spontaneous formation of P80 micelle within 10 ns is consistent with the experimental findings of the spontaneous formation of micelles. The findings of P80 were also consistent with Mobasheri’s article (18).

All the structures SPA and micelle (SPA+ P80) and Micelle-MB were also formed in a nanosecond timeframe, indicating their spontaneous formation. These results align with the observed phenomena in the laboratory. 

Adding P80 to the simulation box containing SPA altered the SPA molecule’s aggregation number. This suggests that the SPA aggregation number depends on the molecules it interacts with and its surroundings and that there is no fixed number. According to the Alargova article, the aggregation number of micelles is influenced by various factors, including the surfactant’s characteristics, temperature, the type and concentration of electrolytes added, and organic additives (22).

MB was located in the shell or hydrophilic part of the Micelle-MB. It was expected because MB is soluble in water (23).

The result of potential energy initially decreasing as the micelle increases in size, followed by a phase of stabilization, is consistent with thermodynamic principles, which state that systems tend to attain a minimal energy level (24). This principle underpins the formation of micelles, where the system minimizes its energy by forming these structures.

Micelles are formed when there is minimal contact of the surfactant’s hydrophobic parts with the aqueous environment (24). This supports the concept that an increasing SASA indicates micelle dissolution in the solvent, and a decrease in the free area on the water surface leads to aggregation. Thus, increasing SASA and having a stable plot means the micelle is dissolved in the solvent. When the free area of ​​the micelle decreases on the water surface, aggregation occurs.

The results show that the Micelle-MB are semi-spherical (ellipsoidal), which, according to the Soares study, MB-loaded polymeric micelles show a spherical shape (25). Also, in the Bharate study, the Transmission Electron Microscopy (TEM) analysis of a Poly (styrene-co-maleic acid) Micelle of MB revealed the presence of a spherical micelle formed (14).

The average energy of MB-MB and P80-Water interactions before and after encapsulation, as shown in (Figure 4), is unchanged, supporting the MBs’ monomer solubilization and self-assembly of P80 surfactants. The rise in energy of SPA-water interactions during encapsulation further demonstrates the hydrophobic nature of the dissolving process. This result confirms the lipophilic property of SPA (26). 

According to the mentioned results and observing the efficiency of CAP treatment, as well as the halving of CAP radiation time, when using Micelle-MB, it is suggested that this project be carried out on a larger scale and other phases of research to fully understand the results of CAP treatment on glioblastoma and its ability to reduce hypoxia during photodynamic therapy should be investigated. 

Table 3 shows the PDI of micelles; in micelles with a concentration of 0.01 mM MB, the nano micelle PDI is in a more suitable range than a concentration of 0.005 MB. This can lead to the conclusion that a higher concentration of MB in the structure confers greater stability. According to a review article, the stability of micelles is also contingent upon the composition and concentration of disrupting agents present in the solution (27). So, the stability of the micelle may change with dye concentration. Also, in higher concentrations of MB, the nano micelle size is smaller. The size of nano micelles, self-assembled structures formed by surfactants, can be influenced by the concentration of the components in their formation. Higher concentrations of dye molecules or other components may lead to the formation of smaller nano micelles due to increased packing density and more efficient self-assembly processes. In the Soares study, Polymeric micelles loaded with MB demonstrated a decrease in particle size (25).

Optimizing the relative amount of surfactants (2/3 SPA and 1/3 P80) in the MD simulation produced favorable laboratory results.

The IC_50_ value of MB in the current study was 27.5 μM. However, the IC_50 _of Micelle-MB was observed to be close to 18 μM, indicating that MB has relatively lower toxicity than Micelle-MB. This finding is consistent with the results of the study conducted by Yan *et al.* regarding the increase in the dye’s toxicity after adding a micellar structure (28).

Based on Figure 6, The relative decline in optical density of DPBF in Micelle-MB was higher than that in MB, suggesting that Micelle-MB may play a more effective role in ROS production than MB. However, other factors may have influenced the results, and further data is needed to draw definitive conclusions. 

It has been predicted that the structure of MB will not be altered due to only brief periods of irradiation. Jesus *et al.*’s investigation demonstrated that the color of the MB solution changed from deep blue to light blue with increasing argon plasma irradiation time. After 120 min of CAP irradiation, the MB solution became colorless (23). Therefore, it is recommended that an alternative probe be used for prolonged periods of high CAP irradiation.

Since MB absorption overlaps with CAP emission in a particular place (600-700 nm), as shown in (Figure 5B), CAP-induced PDT and active species-produced CAP radiation are simultaneously used in this study. 

### Samples synthesized (Phase #2)

The absorption peak of Micelle-MB indicates the influence of the micellar environment on MB’s electronic structure. This could be because the hydrophobic core of micelles creates a different polarity environment compared to water. Encapsulation of MB in micelles likely stabilizes its electronic state, resulting in a higher absorption peak. These results align with Umezawa’s study, suggesting that a micellar environment can stabilize the dye’s electronic state, causing changes in absorption peaks (29).

The spectral overlap and energy transfer mechanisms boost ROS generation, improving MB’s efficacy in PDT. Tian’s research explains how energy transfer enhances photosensitizer efficiency, leading to better ROS generation and therapeutic outcomes in PDT (30). 

The hydrodynamic size of micelles decreases with increasing MB concentration, possibly due to better surfactant packing around MB. MB presence affects the micellar core, leading to tighter assembly. Smaller, uniform micelles enhance drug delivery and reduce side effects. Studies like Hari *et al.*. suggest that modifying micelle size distribution improves drug delivery efficiency (31).

The survival percentage is lower in the Micelle-MB group than in the MB group, indicating enhanced internalization of MB by cells through micellar formulation. Nanoscale size and surface properties of micelles improve MB uptake by interacting with cellular membranes. Micelle-MB exposed cells have higher intracellular MB concentrations, increasing cytotoxic effects. Research by Hari *et al*. (31) shows that micelles enhance interaction with cellular membranes, leading to higher drug concentrations and improved therapeutic efficacy.

The comparison of DPBF+MB and DPBF+Micelle-MB spectra, when subjected to varying irradiation durations, illustrates that Micelle-MB demonstrates a more notable reduction in the DPBF absorption peak at 490 nm. This observation suggests that Micelle-MB produces more singlet oxygen than MB. The enhancement of PDT efficacy by Micelle-MB is probably attributed to its improved solubility, stability, and affinity towards DPBF. These findings are derived from an investigation emphasizing how enhanced solubility, stability, and affinity towards target molecules can result in superior PDT outcomes.

The combination of CAP and Micelle-MB demonstrates superior efficacy in diminishing cell viability compared to CAP with MB or the control group. The potential enhancement of Micelle-MB facilitates its absorption by the cells, resulting in elevated intracellular levels and heightened susceptibility to CAP therapy. This assertion is corroborated by the investigation by Shanmugham *et al.* (33), suggesting that the micellar composition boosts the cellular uptake of capsanthin, leading to escalated intracellular concentrations and heightened cytotoxic repercussions compared to the non-micellar composition.

As the duration of radiation increases, the SYN implies that prolonged irradiation to CAP amplifies the cooperative impacts between CAP and MB. The augmented SYN values suggest that the combination of CAP and MB exhibits heightened efficacy in impeding cell proliferation during extended radiation exposure. This assertion finds reinforcement in the investigation by Shayesteh Ara *et al.* (8), which suggests that incorporating PS into CAP therapy augments the efficacy of cancer treatment.

### Plasma-induced PDT (Phase #3)

The inhibitory effect of CAP-mediated photodynamic action on the glioblastoma cell line (U87-MG) increases with exposure time, as observed from the computation of the synergistic ratio (Table 4) and ET_50_ of CAP treatment. These findings agree with a recent investigation by Shayesteh Ara *et al.* (8).

With the increase in CAP irradiation time, the percentage of cell survival also decreases significantly. However, there is no significant relationship between 30 sec and 60 sec in the MB group. A study found that a low dose of CAP can increase the proliferation of endothelial cells due to the production of ROS (34). Also, Moniruzzaman *et al.* considered a threshold for needed ROS to induce apoptosis. They report that in the preliminary times of ROS formation, its effect is lower than the threshold and insufficient for induction apoptosis (35). Considering the results of these two studies, it can be assumed that in the MB group at times 30 sec and 60 sec, the ROS received by the cell was not sufficient and did not cause apoptosis, but in the Micelle-MB group, it is predicted that the cells uptake micelles more, and induced photodynamic effects are more involved in ROS production. In the study of Shayesteh Ara *et al.*, the time required for CAP irradiation (Plasma jet current 290–350 mA) at a concentration of 10 μM Indocyanine green (ICG) to kill 50% of U87-MG cells was reported to be 96 sec (8); in the present study; this time was obtained in the concentration of 15 μM MB, 40 sec in the Micelle-MB group, 86 sec in the MBgroup. This difference may be attributed to variations in flow rate, concentration, and the specific type of PS employed. From the comparison of the results of PS receiving groups in two studies, considering that both CAP irradiation was done at IC_30_ of the PS, it can be predicted that MB was more effective than ICG during CAP irradiation.

Figure 7 illustrates that the CAP ET_50_ of the MB group is roughly 86 sec, while that of the Micelle-MB group is approximately 40 sec. Therefore, micelles can halve the irradiation time at the selected MB concentration for the same survival percentage. 

Utilizing micelle has proven to be efficacious in improving treatment circumstances while minimizing the amount of PS employed. Furthermore, the results of Bharate ‘s study, which investigated the influence of photodynamics using Micelle with MB in esophageal cancer, are congruent with our findings about using micelle-MB in the induction of PDT (14). A preliminary investigation was carried out to examine the effects of the MB-loaded polyacrylamide nanoparticles on rat C6 glioma tumor cells, and effective photodynamic outcomes were observed. The inclusion of MB within the nanoparticles is expected to reduce the interaction between this PS and the surrounding biological environment, thereby enabling its administration throughout the body (36). Also, a study concluded that adding a polymer micelle structure to chlorin e6 dye in photodynamic therapy increases the cell’s absorption of the dye and thus increases the treatment’s effectiveness (37).

Micellar nanostructures provide many advantages, the most important of which are dimensional control ability, increased photochemical efficiency, the release of medicinal agents with optimum frequency and dose, and the ability to accumulate in tumors selectively after intravenous injection (38, 39). Junqueira’s results show that the photochemical properties of MB+ can be altered due to the equilibrium between the monomer and dimer states induced by the micelles (40). Micellar stability can be enhanced by controlling hydrophobic effects within the inner micellar structure by implementing chemical cross-links. Increasing the hydrophobicity of supramolecular polymeric micelles and improving their stability can enhance their cellular uptake and ability to induce apoptosis in cancer cells (41). Hydrogen bonding frequently arises within the hydrophobic segments of two copolymers, conferring stability to the core of Polymeric micelles and consequently enhancing their overall stability (42). According to what was said about other micelles, these things may also be acceptable for micelle-MB. So, enhancing PS stability, enhancing photochemical capabilities, decreasing aggregation of PS, hydrogen bonds forming between the surfactants in a system with many surfactants, and increasing the system’s stability were factors that informed the decision to use micelle for this study.

**Table 1:** Parameters such as time and the number of molecules for creating a simulation box for molecular dynamics simulation

Simulation phase	Time (ns)	Number of molecule(s)			
		P80	SPA	MB	CG Water
P80 Micelle	10	60	-	-	9611
SPA Micelle	100	-	60	-	3208
P80 with MB	10	60	-	1	9883
SPA and P80 Micelle	100	60	60	-	9329
SPA and P80 Micelle	100	60	90	-	9328
Micelle-MB	100	60	90	10	9219

MB: Methylene blue

**Table 2:** Average radius of gyration (Rg) values calculated via molecular dynamics, showing the core, shell, and complete micelle

R_g _(nm)		
Core radius	Shell thickness	Micelle (Core+Shell)
3.7	2.03	5.73

SPA: Span80

**Table 3:** Results of PDI and size distribution analysis based on different concentrations of MB in micelles with the same concentration of SPA and P80

MB concentration (mM)	SPA concentration (mM)	P80 concentration (mM)	Z Average (nm)	PDI
0.005	1.5	1	22.7	0.606
0.01	1.5	1	18.1	0.127

PDI: Polydispersity index; MB: Methylene blue; SPA: Span80

**Table 4:** SYN of different durations of CAP irradiation in two groups: MB and Micelle-MB

PS	Time (s)	Synergism rate ± standard deviation	mean Synergism rate ± standard deviation
Micelle-MB	30	1.08 ± 0.24	1.54±0.5
	60	1.56 ± 0.11	
	90	1.98 ± 0.71	
MB	30	1.26 ± 0.62	1.41± 0.7
	60	1.28 ± 0.37	
	90	1.70 ± 0.65	

SYN: Synergistic; CAP: Cold atmospheric plasma; MB: Methylene blue

## Conclusion

This investigation utilized the technique of MD modeling to develop a novel nanostructure employing optimized Micelles-MB dye. The purpose was to heighten the efficacy of the impacts of the induced photodynamics of CAP on human glioblastoma cell lines. Nanostructure preparation in a laboratory-based on computational nanotechnology-based techniques (MD modeling) facilitated the design and preparation of an efficient photosensitizer. Our findings suggested that the application of CAP on U87-MG cells diminished their survival rate, thereby expanding the effectiveness of the treatment through the selection of the appropriate timing and concentration of micelle-MB. Notably, the Micelle-MB exhibited a lower survival rate than the MB group, signifying an improvement in the treatment. The treatment efficacy was enhanced by two distinct processes, namely, the direct action of the RONS produced by the CAP and the induced photodynamic effect arising from the blue flame of the CAP. Moreover, MD simulation facilitated precise prediction of crucial factors such as drug (MB) loading by the carrier (micelle), drug location within the micelle, its size and shape, and micelle formation and thermodynamic equilibrium. One limitation of this project is the failure to convert the concentrations utilized in laboratory experiments into values suitable for simulation purposes.

## Declaration

In the process of optimizing the article, we used AI tools exclusively for grammar correction (Grammarly and paraphrasing tool Quillbot). These tools are used to increase the clarity and readability of the text and ensure that the content is presented in a coherent and professional manner. All the basic content, analysis and conclusions are the main work of the authors.
